# Predictive model for prolonged hospital stay risk after gastric cancer surgery

**DOI:** 10.3389/fonc.2024.1382878

**Published:** 2024-08-06

**Authors:** Xiaochun Zhang, Xiao Wei, Siying Lin, Wenhao Sun, Gang Wang, Wei Cheng, Mingyue Shao, Zhengming Deng, Zhiwei Jiang, Guanwen Gong

**Affiliations:** ^1^ The First Clinical College of Nanjing University of Chinese Medicine, Nanjing, Jiangsu, China; ^2^ Department of General Surgery, Affiliated Hospital of Nanjing University of Chinese Medicine, Nanjing, Jiangsu, China

**Keywords:** gastric cancer, PLOS, nomogram, rehabilitation, perioperative period

## Abstract

**Background:**

Prolonged postoperative hospital stay following gastric cancer (GC) surgery is an important risk factor affecting patients’ mood and increasing complications. We aimed to develop a nomogram to predict risk factors associated with prolonged postoperative length of stay (PLOS) in patients undergoing gastric cancer resection.

**Methods:**

Data were collected from 404 patients. The least absolute shrinkage and selection operator (LASSO) was used for variable screening, and a nomogram was designed. The nomogram performance was evaluated by the area under the receiver operating characteristic curve (AUC). The consistency between the predicted and actual values was evaluated via a calibration map, and the clinical application value was evaluated via decision curve analysis (DCA) and clinical impact curve analysis (CICA).

**Results:**

A total of 404 patients were included in this study. Among these patients, 287 were assigned to the training cohort, and 117 were assigned to the validation cohort. According to the PLOS quartile distance, 103 patients were defined as having prolonged PLOS. LASSO regression and logistic multivariate analysis revealed that 4 clinical characteristics, the neutrophil–lymphocyte ratio (NLR) on postoperative day one, the NLR on postoperative day three, the preoperative prognostic nutrition index and the first time anal exhaust was performed, were associated with the PLOS and were included in the construction of the nomogram. The AUC of the nomogram prediction model was 0.990 for the training set and 0.983 for the validation set. The calibration curve indicated good correlation between the predicted results and the actual results. The Hosmer-Lemeshow test revealed that the P values for the training and validation sets were 0.444 and 0.607, respectively, indicating that the model had good goodness of fit. The decision curve analysis and clinical impact curve of this model showed good clinical practicability for both cohorts.

**Conclusion:**

We explored the risk factors for prolonged PLOS in GC patients via the enhanced recovery after surgery (ERAS) program and developed a predictive model. The designed nomogram is expected to be an accurate and personalized tool for predicting the risk and prognosis of PLOS in GC patients via ERAS measures.

## Introduction

Gastric cancer (GC) is currently the fifth most prevalent cancer globally, accounting for the fourth-highest number of cancer-related fatalities. Despite the observed decline in GC incidence and the introduction of various prevention, screening, and treatment initiatives worldwide, the situation remains concerning in Asia, particularly in East Asia, where GC continues to represent a significant global health concern (1). In China, GC ranks third in incidence and mortality among all forms of malignant tumors (2). Radical resection is a crucial intervention for increasing the survival rate of patients affected by both early and advanced gastric cancer (3). Nonetheless, it is imperative not to disregard the issue of perioperative complications. Postoperative complications occur frequently among gastric cancer patients, and several studies have indicated that the incidence of such complications can range from 20% to 46% (4). The duration of postoperative hospitalization not only influences patient safety and contentment but also provides hospitals with a means to enhance medical care, improve quality, and manage costs. Prolonged hospitalization following surgical procedures contributes to patient distress and increases vulnerability to infections, deep vein thrombosis, and other complications. In addition, long postoperative stays typically indicate subpar care quality and exorbitant medical expenses. Thus, finding effective strategies to mitigate complications, reduce the duration of hospital stay, and improve the short-term prognosis of patients has emerged as a primary challenge for clinicians and medical institutions.

The fast-track surgery concept, initially introduced by Henrik Kehlet and advanced by the Enhanced Recovery After Surgery (ERAS) Society, has experienced substantial growth and revolutionized the surgical field (5). ERAS is widely recognized for its ability to mitigate the body’s stress response, facilitate prompt functional recovery, and considerably reduce patients’ prolonged postoperative length of stay (PLOS), leading to cost savings and increased medical efficiency (6). A composite of surgical quality metrics, including surgical intent, resection margin status, lymph node sampling adequacy, intraoperative and postoperative complications, reinterventions, intensive care unit (ICU) admissions, length of hospital stay, readmissions, and mortality, has been proposed (7). The fast-track surgery concept is a new concept in GC surgery and represents a composite of surgical quality indicators that are strongly associated with improved long-term survival (8).

Recent studies have indicated that factors such as preoperative neoadjuvant chemotherapy, surgical approach (minimally invasive surgery), time to first exhaust after surgery, specific surgical techniques, and patient compliance can influence the ERAS protocol in GC patients (9). Although nomogram prediction models have been widely utilized for postoperative outcomes, an ERAS management model to establish a prediction model for the risk of prolonged hospitalization after GC surgery is lacking. Robot-assisted radical gastrectomy has been performed at Jiangsu Province Hospital of Traditional Chinese Medicine since 2018. Under the guidance of Professor Jiang Zhiwei’s team, more than 1500 cases of gastric cancer have been treated with accurate surgical anatomy and minimal trauma. Consequently, this study aimed to analyze the clinical characteristics of patients who underwent GC surgery with an ERAS management approach, develop a nomogram to predict prolonged postoperative hospital stays in patients with GC, identify risk factors associated with longer PLOS, and provide individualized guidance for patients, as well as a foundation for the effective implementation of ERAS.

## Materials and methods

### Study population

**Figure 1:**
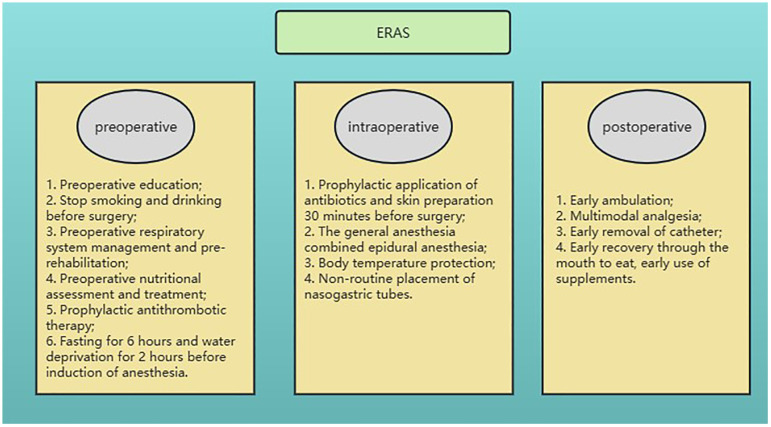
Perioperative enhanced recovery after surgery.

A total of 404 patients who underwent gastrectomy for GC at the Jiangsu Province Hospital of Chinese Medicine (Affiliated Hospital of Nanjing University of Chinese Medicine) between November 1, 2018, and November 31, 2022, were selected retrospectively for this study. To obtain reliable model prediction values, the model randomly divided the dataset 100 times at a ratio of 7:3, and 100 prediction results were obtained. There were 287 and 117 patients in each group. This study was approved by the Ethics Committees of the Affiliated Hospital of Nanjing University of Chinese Medicine and was conducted in accordance with the Helsinki Declaration. Informed consent was obtained from all patients included in the study. The ERAS management pathway was implemented for all patients and included preoperative prerehabilitation, intraoperative temperature protection, multimodal analgesia, early postoperative ambulation, early nutritional support, and other interventions (**Figure 1**).

Demographic and clinical parameters, including sex, age, body mass index (BMI), gastroscopy pathology, neoadjuvant chemotherapy, American Society of Anaesthesiologists (ASA) score, surgical method, surgery duration, and preoperative and postoperative laboratory results (including white blood cell (WBC) count, hemoglobin (Hb) level, neutrophil percentage, albumin level (ALB), neutrophil-lymphocyte ratio (NLR), C-reactive protein (CRP)-ALB ratio (CAR), total protein (TP), prealbumin (PAB), transferrin (TRF) and prognostic nutritional index (PNI)), tumor characteristics (T stage, N stage, M stage, AJCC stage, tumor invasion depth), pain score on the first day after surgery and first time to anal exhaust), and postoperative complication occurrence (such as bleeding, anastomotic fistula, anastomotic stenosis, duodenal stump fistula, pulmonary complications, pancreatic fistula, lymphatic fistula, surgical incision infection, and intestinal obstruction), were collected. The classification and number of related postoperative complications are detailed in **Table 1**.

**Table 1:** Classification and number of postoperative complications.

Classification of complications	Name of Complication	Number of patients
Gastrointestinal-related complications	Anastomotic fistula	8
	Abdominal pelvic infection	4
	Chylous fistula	2
	Bleeding	3
	Mechanical intestinal obstruction	3
	Paralytic ileus	4
	diarrhea	6
	Pancreatic fistula	1
	Delayed gastric emptying	2
Incision-related complications	Delayed incision healing	1
	Drainage site infection	2
Respiratory complications	Postoperative pneumonia	3

### Inclusion and exclusion criteria

The inclusion criteria for patients were as follows (1): aged 18–85 years (2); diagnosed with GC by postoperative histopathology; and (3) underwent radical gastrectomy for GC.

The exclusion criteria were as follows (1): died after surgery (2); other organ resections were performed (3); incomplete data were available (4); refusal or inability to cooperate (5); other malignant tumors or distant metastases and palliative surgery; and (6) emergency surgery, such as intestinal obstruction and bleeding, was performed.

### Surgical methods

All operations were performed by the same surgeon. After successful anaesthesia, all patients were placed in the supine position with their legs apart, head elevated, feet lowered, and left side elevated higher than the right. The trocar layout used for robotic surgery was the same as that used in the literature (10). The conventional 5-port method was used for laparoscopic surgery, and a median upper abdominal incision was used for open surgery. Surgical resection and lymph node dissection were performed according to the Japanese Gastric Cancer Treatment Guidelines (5th edition) (11). A small upper abdominal incision and hand-assisted gastrointestinal anastomosis were used for digestive tract reconstruction in the robot-assisted gastrectomy (RAG) and laparoscopy-assisted gastrectomy (LAG) groups. The distal stomach and the whole stomach were reconstructed via Roux-en-Y digestive tract reconstruction, and the proximal stomach was reconstructed via direct anastomosis between the posterior wall of the stomach and the oesophagus.

### Model construction and statistical analysis

The participants were divided into training and validation groups via the cluster random sampling method at a 7:3 ratio. Data analysis was conducted via R 4.2.2 and SPSS 26.0 software. The age variable was transformed via X-tile software, categorizing individuals as either <65 years old or ≥65 years old. Normally distributed continuous variables are expressed as the mean ± standard deviation (SD) and were analyzed via Student’s t test. Skewed continuous variables were characterized via median values (25th percentile, 75th percentile) and tested via the Mann-Whitney U test. Count data are presented as rates or percentages, and comparisons between two groups were analyzed via the weighted chi-square test or Fisher’s exact test.

The prescreening data were normalized, and least absolute shrinkage and selection operator (LASSO) regression analysis was conducted via the ‘glmnet’ package to identify predictors. Multivariate logistic regression analysis was employed to develop a prediction model, and the ‘rms’ package was used to construct a nomogram. A receiver operating characteristic (ROC) curve was generated to assess the model’s predictive performance, and the area under the curve (AUC) was calculated through ROC curve analysis. The clinical applicability of the nomogram was verified via decision curve analysis (DCA) and clinical impact curve analysis (CICA). A statistically significant level of P<0.05 was considered.

## Results

### Clinical data

**Figure 2:**
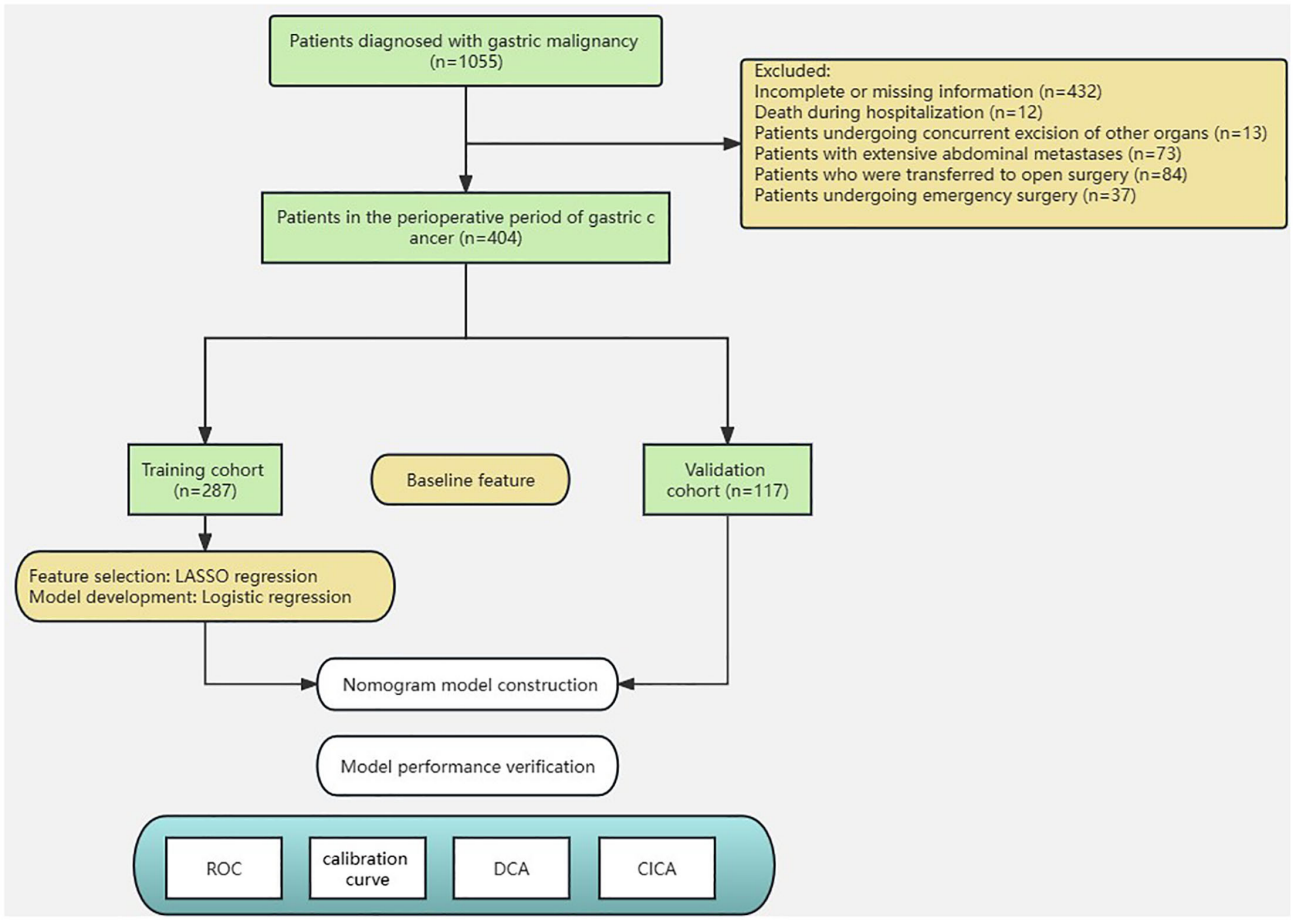
Flowchart of the study design.

The study included a total of 404 patients with gastric cancer. Among these patients, 287 were randomly assigned to the training set, whereas the remaining 117 were assigned to the validation set. (**Figure 2**). To assess the impact of the PLOS, we examined its distribution for normality. However, we discovered that the PLOS data did not adhere to a normal distribution (*p* < 0.001). Consequently, the PLOS was determined through the use of median and quartile intervals. Specifically, the median PLOS was 8 days, whereas the 75th percentile PLOS for the entire sample was 13 days. Therefore, in this study, PLOS exceeding the 75th percentile was categorized as prolonged PLOS (12–14). The baseline characteristics of the two groups were balanced, as shown in **Table 2**.

**Table 2:** Basic characteristics of patients in the training and validation groups.

Characteristic	All patients (*n*=404)	Training set (*n*=287)	Validation set (*n*=117)	*p* value
Gender, *n* (%)				0.7849
Male	299 (74.01)	214 (74.56)	85 (72.65)	
Female	105 (25.99)	73 (25.44)	32 (27.35)	
Age, *n* (%)				0.5558
<65	194 (48.02)	141 (49.13)	53 (45.30)	
≥65	210 (51.98)	146 (50.87)	64 (54.70)	
BMI, kg/m^2^, *n* (%)				0.4153
<18	19 (4.70)	12 (4.18)	7 (5.98)	
18–28	351 (86.88)	248 (86.41)	103 (88.03)	
>28	34 (8.42)	27 (9.41)	7 (5.98)	
Primary diseases, *n* (%)				0.4588
No	218 (53.96)	151 (52.61)	67 (57.26)	
Yes	186 (46.04)	136 (47.39)	50 (42.74)	
Gastroscopic pathology, *n* (%)				0.7369
Adenocarcinoma	314 (77.72)	223 (77.70)	91 (77.78)	
Signet ring cell carcinoma	63 (15.59)	44 (15.33)	19 (16.24)	
Carcinoma in situ	24 (5.94)	17 (5.92)	7 (5.98)	
Mixed type	3 (0.74)	3 (1.05)	0 (0.00)	
Neoadjuvant chemotherapy, *n* (%)				0.7054
No	248 (61.39)	174 (60.63)	74 (63.24)	
Yes	156 (38.61)	113 (39.37)	43 (36.75)	
ASA score, *n* (%)				0.7624
1–2	277 (68.56)	195 (67.94)	82 (70.09)	
3–4	127 (31.44)	92 (32.06)	35 (29.91)	
Operation method, *n* (%)				0.2505
Open surgery	102 (25.25)	75 (26.13)	27 (23.08)	
Laparoscope	178 (44.06)	119 (41.46)	59 (50.43)	
Robot	124 (30.69)	93 (32.40)	31 (26.50)	
Operation time, min, *n* (%)				0.5328
< 180	48 (11.88)	36 (12.54)	12 (10.26)	
180–300	270 (66.83)	187 (65.16)	83 (70.94)	
>300	86 (21.29)	64 (22.30)	22 (18.80)	
Pre WBC,/L, *n* (%)				0.8601
<4.0x10^9^	16 (3.96)	12 (4.18)	4 (3.42)	
(4.0~10.0) ×10^9^	368 (91.09)	260 (90.59)	108 (92.31)	
>10.0x10^9^	20 (4.95)	15 (5.23)	5 (4.27)	
Pre Hb, g/L, *n* (%)				0.8549
<90	55 (13.61)	38 (13.24)	17 (14.53)	
≥90	349 (86.39)	249 (86.76)	100 (85.47)	
Pre Neutrophil percentage, %, *n* (%)				0.4284
<40	18 (4.46)	15 (5.23)	3 (2.56)	
40–80	369 (91.34)	259 (90.24)	110 (94.02)	
>80	17 (4.21)	13 (4.53)	4 (3.42)	
Pre ALB, g/L, *n* (%)				0.6576
<30	7 (1.73)	6 (2.09)	1 (0.85)	
≥30	397 (98.27)	281 (97.91)	116 (99.15)	
Pre NLR, (median [IQR])	2.150 [1.438, 3.140]	2.150 [1.45, 2.985]	2.180 [1.405, 3.810]	0.7036
Pre CAR, (median [IQR])	0.230 [0.150, 0.320]	0.23 [0.15, 0.31]	0.25 [0.145, 0.32]	0.8583
Pre TP, (median [IQR])	65.600 [59.823, 70.158]	65.290 [59.590, 69.920]	66.360 [60.890, 70.810]	0.1313
Pre PAB, (median [IQR])	144.720 [112.778, 178.895]	145.620 [111.550, 178.380]	141.050 [113.760, 179.950]	0.8835
Pre TRF, (median [IQR])	2.525 [2.160, 2.790]	2.500 [2.135, 2.760]	2.570 [2.250, 2.860]	0.1047
Pre PNI, (median [IQR])	49.235 [47.328, 51.458]	49.370 [47.250, 51.485]	49.100 [47.470, 51.300]	0.7015
Pod1 WBC,/L, *n* (%)				0.7448
<4.0×10^9^	1 (0.25)	1 (0.35)	0 (0.00)	
(4.0~10.0) ×10^9^	165 (40.84)	119 (41.46)	46 (39.32)	
>10.0×10^9^	238 (58.91)	167 (58.19)	71 (60.68)	
Pod1 Hb, g/L, *n* (%)				0.7509
<90	57 (14.11)	42 (14.63)	15 (12.82)	
≥90	347 (85.89)	245 (85.37)	102 (87.18)	
Pod1 Neutrophil percentage, %, *n* (%)				0.1147
<40	1 (0.25)	1 (0.35)	0 (0.00)	
40–80	60 (14.85)	49 (17.07)	11 (9.40)	
>80	343 (84.90)	237 (82.58)	106 (90.60)	
Pod1 ALB, g/L, *n* (%)				0.0995
<30	24 (5.94)	13 (4.53)	11 (9.40)	
≥30	380 (94.06)	274 (95.47)	106 (90.60)	
Pod1 NLR, (median [IQR])	11.965[10.06, 14.42]	11.73[10.00, 14.52]	12.20[10.36, 14.385]	0.3286
Pod1 CAR, (median [IQR])	0.955 [0.79, 1.08]	0.96 [0.78, 1.07]	0.95 [0.80, 1.09]	0.6485
Pod1 TP, (median [IQR])	58.230 [54.383, 61.885]	58.390 [54.520, 62.360]	57.600 [53.930, 60.580]	0.1816
Pod1 PAB, (median [IQR])	134.350 [114.547, 153.140]	134.140 [115.005, 152.895]	135.490 [114.020, 154.990]	0.9685
Pod1 TRF, (median [IQR])	2.110 [1.940, 2.400]	2.120 [1.940, 2.400]	2.090 [1.950, 2.400]	0.9024
Pod1 PNI, (median [IQR])	40.830 [38.288, 43.210]	41.040 [38.255, 43.210]	40.570 [38.400, 43.080]	0.9906
Pod3 WBC,/L, n (%)				0.4926
(4.0~10.0) ×10^9^	281 (69.55)	203 (70.73)	78 (66.67)	
>10.0×10^9^	123 (30.45)	84 (29.27)	39 (33.33)	
Pod3 Hb, g/L, n (%)				0.2643
<90	117 (28.96)	78 (27.18)	39 (33.33)	
≥90	287 (71.04)	209 (72.82)	78 (66.67)	
Pod3 Neutral percentage, %, n (%)				0.4550
40–80	312 (77.23)	225 (78.40)	87 (74.36)	
>80	92 (22.77)	62 (21.60)	30 (25.64)	
Pod3 ALB, g/L, n (%)				0.2341
<30	93 (23.02)	61 (21.25)	32 (27.35)	
≥30	311 (76.98)	226 (78.75)	85 (72.65)	
Pod3 NLR, (median [IQR])	6.845 [4.492, 8.688]	6.900 [4.325, 8.770]	6.760 [4.970, 8.230]	0.6880
Pod3 CAR, (median [IQR])	0.715 [0.458, 0.980]	0.700 [0.435, 0.970]	0.780 [0.520, 0.990]	0.1620
Pod3 TP, (median [IQR])	62.200 [58.940, 65.150]	61.990 [58.900, 64.970]	62.550 [59.280, 65.400]	0.1477
Pod3 PAB, (median [IQR])	153.425 [128.675, 177.947]	153.010 [128.965, 178.050]	155.980 [127.130, 176.920]	0.9918
Pod3 TRF, (median [IQR])	2.110 [1.870, 2.420]	2.120 [1.880, 2.425]	2.070 [1.830, 2.420]	0.4540
Pod3 PNI, (median [IQR])	48.250 [43.425, 52.470]	48.690 [43.560, 52.555]	46.430 [43.400, 51.640]	0.1528
T stage, *n* (%)				0.4989
T1	100 (24.75)	66 (23.00)	34 (29.06)	
T2	50 (12.38)	37 (12.89)	13 (11.11)	
T3	194 (48.02)	138 (48.08)	56 (47.86)	
T4	60 (14.85)	46 (16.03)	14 (11.97)	
N stage, *n* (%)				0.0654
N0	158 (39.11)	105 (36.59)	53 (45.30)	
N1	64 (15.84)	54 (18.82)	10 (8.55)	
N2	76 (18.81)	53 (18.47)	23 (19.66)	
N3	106 (26.24)	75 (26.13)	31 (26.50)	
M stage, *n* (%)				0.4894
M0	398 (98.51)	284 (98.95)	114 (97.44)	
M1	6 (1.49)	3 (1.05)	3 (2.56)	
AJCC stage, *n* (%)				0.3821
I	112 (27.72)	75 (26.13)	37 (31.62)	
II	120 (29.70)	88 (30.66)	32 (27.35)	
III	161 (39.85)	118 (41.11)	43 (36.75)	
IV	11 (2.72)	6 (2.09)	5 (4.27)	
Depth of infiltration, *n* (%)				0.1207
mucosal layer	29 (7.18)	15 (5.23)	14 (11.97)	
submucosa	55 (13.61)	39 (13.59)	16 (13.68)	
muscle layer	92 (22.77)	66 (23.00)	26 (22.22)	
serous membrane layer	228 (56.44)	167 (58.19)	61 (52.14)	
Pod1 pain score, *n* (%)				0.7608
1–3	262 (64.85)	188 (65.51)	74 (63.25)	
4–6	129 (31.93)	89 (31.01)	40 (34.19)	
7–10	13 (3.22)	10 (3.48)	3 (2.56)	
Exhaust time, h (median [IQR])	87.000 [68.750, 120.000]	87.000 [68.000, 120.000]	89.000 [72.000, 126.000]	0.2498
Complication, *n* (%)				0.5051
No	365 (90.35)	257 (89.55)	108 (92.31)	
Yes	39 (9.65)	30 (10.45)	9 (7.69)	
Prolonged PLOS, *n* (%)				0.5015
No	301 (74.50)	217 (75.61)	84 (71.79)	
Yes	103 (25.50)	70 (24.39)	33 (28.21)	

**Table 3** presents a comparison of the baseline characteristics between the two groups: prolonged PLOS and nonprolonged PLOS. The results revealed that patients in the prolonged PLOS group were more likely to be older (χ*^2^ =* 5.712, *p*=0.0229) and to have a greater incidence of primary disease (χ*^2^ =* 8.300, *p*=0.0057), an elevated ASA score (χ*^2^ =* 28.263, *p*<0.0001), a longer exhaust time (*Z*=-10.286, *p*<0.0001), a greater pre NLR (*t*=-2.353, *p*=0.020), a lower pre TRF (*Z*=-7.118, *p*<0.0001), a lower pre PNI (*Z*=-11.070, *p*<0.0001), a greater postoperative day one (Pod1) NLR (*Z*=-15.563, *p*<0.0001), a greater Pod1 CAR (*Z*=-10.234, *p*<0.0001), a lower Pod1 TRF (*Z*=-11.136, *p*<0.0001), a lower Pod1 PNI (*Z*=-14.348, *p*<0.0001), a greater Pod3 WBC (χ*^2^ =* 28.820, *p*<0.0001), a lower Pod3 ALB (χ*^2^ =* 17.191, *p*=0.0001), a greater Pod3 NLR (*Z*=-9.985, *p*<0.0001), a greater Pod3 CAR (*Z*=-9.643, *p*<0.0001), a lower Pod3 TRF (*Z*=-14.099, *p*<0.0001) and a lower Pod3 PNI (*Z*=-9.835, *p*<0.0001) than those in the nonprolonged PLOS group. The incidence of complications (χ*^2^ =* 48.716, *p*<0.0001) was higher in the prolonged PLOS group. Additionally, significant differences in surgical methods were observed between the two groups of patients: robotic surgery was associated with a lower probability of prolonged PLOS than were open surgery (χ*^2^ =* 21.914, *p*<0.0001) and laparoscopic surgery (χ*^2^ =* 30.900, *p*<0.0001). However, no significant difference was found in the delay in the PLOS between patients who underwent open surgery and those who underwent laparoscopic surgery (χ*^2^ =* 0.348, *p*=0.555). Furthermore, the Pod1 pain score differed significantly between the two patient groups. Patients with pain scores of 1–3 were less likely to have prolonged PLOS than those with pain scores of 4–6 (*χ^2^ =* 11.767, *p*<0.001), and patients with pain scores of 4–6 were less likely to have prolonged PLOS than those with pain scores of 7–10 (*χ* ^2^ = 14.400, *p*<0.001).

**Table 3:** Comparison of general information between the prolonged and nonprolonged PLOS groups.

Characteristic	All patients (*n*=404)	Nonprolonged group (*n*=301)	Prolonged group (*n*=103)	*p* value
Gender, *n* (%)				0.3316
Male	299 (74.01)	227 (75.42)	72 (69.90)	
Female	105 (25.99)	74 (24.58)	31 (30.10)	
Age, *n* (%)				0.0229
<65	194 (48.02)	155 (51.50)	39 (37.86)	
≥65	210 (51.98)	146 (48.50)	64 (62.14)	
BMI, kg/m^2^, *n* (%)				0.8013
<18	19 (4.70)	13 (4.32)	6 (5.83)	
18–28	351 (86.88)	262 (87.04)	89 (86.41)	
>28	34 (8.42)	26 (8.64)	8 (7.77)	
Primary diseases, *n* (%)				0.0057
No	218 (53.96)	175 (58.14)	43 (41.75)	
Yes	186 (46.04)	126 (41.86)	60 (58.25)	
Gastroscopic pathology, *n* (%)				0.4600
Adenocarcinoma	314 (77.72)	230 (76.41)	84 (81.55)	
Signet ring cell carcinoma	63 (15.59)	48 (15.95)	15 (14.56)	
Carcinoma in situ	24 (5.94)	21 (6.98)	3 (2.91)	
Mixed type	3 (0.74)	2 (0.66)	1 (0.97)	
Neoadjuvant chemotherapy, *n* (%)				0.9491
No	248 (61.39)	184(61.13)	64 (62.14)	
Yes	156 (38.61)	117 (38.87)	39 (37.86)	
ASA score, *n* (%)				<0.0001
1–2	277 (68.56)	228 (75.75)	49 (47.57)	
3–4	127 (31.44)	73 (24.25)	54 (52.43)	
Operation method, *n* (%)				<0.0001
Open surgery	102 (25.25)	70 (23.26)	32 (31.07)	
laparoscope	178 (44.06)	116 (38.54)	62 (60.19)	
Robot	124 (30.69)	115 (38.21)	9 (8.74)	
Operation time, min, *n* (%)				0.4424
<180	48 (11.88)	38 (12.62)	10 (9.71)	
180–300	270 (66.83)	203 (67.44)	67 (65.05)	
>300	86 (21.29)	60 (19.93)	26 (25.24)	
Pre WBC,/L, *n* (%)				0.4627
<4.0x10^9^	16 (3.96)	10 (3.32)	6 (5.83)	
(4.0~10.0)x10^9^	368 (91.09)	277 (92.03)	91 (88.35)	
>10.0x10^9^	20 (4.95)	14 (4.65)	6 (5.83)	
Pre Hb, g/L, *n* (%)				1
<90	55 (13.61)	41(13.62)	14 (13.59)	
≥90	349 (86.39)	260 (86.38)	89 (86.41)	
Pre Neutrophil percentage, %, *n* (%)				0.7028
<40	18 (4.46)	14 (4.65)	4 (3.88)	
40–80	369 (91.34)	273 (90.70)	96 (93.20)	
>80	17 (4.21)	14 (4.65)	3 (2.91)	
Pre ALB, g/L, *n* (%)				0.8033
<30	7 (1.73)	6 (1.99)	1 (0.97)	
≥30	397 (98.27)	295 (98.01)	102 (99.03)	
Pre NLR, (median [IQR])	2.150 [1.438, 3.140]	2.12[1.44, 2.93]	2.52[1.41, 4.39]	0.0200
Pre CAR, (median [IQR])	0.230 [0.150, 0.320]	0.23[0.15, 0.31]	0.23[0.11, 0.33]	0.5310
Pre TP, (median [IQR])	65.600 [59.823, 70.158]	65.110 [59.600, 69.820]	66.820 [60.990, 70.785]	0.0992
Pre PAB, (median [IQR])	144.720 [112.778, 178.895]	146.410 [113.760, 178.880]	137.730 [110.465, 180.160]	0.4085
Pre TRF, (median [IQR])	2.525 [2.160, 2.790]	2.610 [2.260, 2.940]	2.280 [1.950, 2.555]	<0.0001
Pre PNI, (median [IQR])	49.235 [47.328, 51.458]	49.960 [48.300, 51.950]	45.970 [43.085, 48.130]	<0.0001
Pod1 WBC,/L, *n* (%)				0.7696
<4.0x109	1 (0.25)	1 (0.33)	0 (0.00)	
(4.0~10.0)x10^9^	165 (40.84)	121 (40.20)	44 (42.72)	
>10.0x10^9^	238 (58.91)	179 (59.47)	59 (57.28)	
Pod1 HB, g/L, *n* (%)				0.1932
<90	57 (14.11)	38 (12.62)	19 (18.45)	
≥90	347 (85.89)	263 (87.38)	84 (81.55)	
Pod1 Neutrophil percentage, %, *n* (%)				0.8234
<40	1 (0.25)	1 (0.33)	0 (0.00)	
40–80	60 (14.85)	44 (14.62)	16 (15.53)	
>80	343 (84.90)	256 (85.05)	87 (84.47)	
Pod1 ALB, g/L, *n* (%)				0.7651
<30	24 (5.94)	19 (6.31)	5 (4.85)	
≥30	380 (94.06)	282 (93.69)	98 (95.15)	
Pod1 NLR, (median [IQR])	11.965[10.055, 14.423]	11.18[9.58, 13.04]	18.98[14.94, 21.46]	<0.0001
Pod1 CAR, (median [IQR])	0.955 [0.790, 1.080]	0.88[0.76, 1.01]	1.10[0.95, 1.33]	<0.0001
Pod1 TP, (median [IQR])	58.230 [54.383, 61.885]	58.000 [54.360, 61.710]	59.000 [54.610, 62.720]	0.2193
Pod1 PAB, (median [IQR])	134.350 [114.547, 153.140]	130.880 [114.390, 151.720]	141.150 [115.250, 155.600]	0.0836
Pod1 TRF, (median [IQR])	2.110 [1.940, 2.400]	2.230 [2.020, 2.500]	1.890 [1.680, 2.020]	<0.0001
Pod1 PNI, (median [IQR])	40.830 [38.288, 43.210]	42.320 [40.130, 43.910]	36.450 [34.565, 37.775]	<0.0001
Pod3 WBC,/L, n (%)				<0.0001
(4.0~10.0) ×10^9^	281 (69.55)	231 (76.74)	50 (48.54)	
>10.0×10^9^	123 (30.45)	70 (23.26)	53 (51.46)	
Pod3 Hb, g/L, n (%)				0.5015
<90	117 (28.96)	84 (27.91)	33 (32.04)	
≥90	287 (71.04)	217 (72.09)	70 (67.96)	
Pod3 Neutral percentage, %, n (%)				0.4072
40–80	312 (77.23)	236 (78.41)	76 (73.79)	
>80	92 (22.77)	65 (21.59)	27 (26.21)	
Pod3 ALB, g/L, n (%)				0.0001
<30	93 (23.02)	54 (17.94)	39 (37.86)	
≥30	311 (76.98)	247 (82.06)	64 (62.14)	
Pod3 NLR, (median [IQR])	6.845 [4.492, 8.688]	5.820 [3.830, 7.900]	8.740 [7.260, 10.405]	<0.0001
Pod3 CAR, (median [IQR])	0.715 [0.458, 0.980]	0.630 [0.410, 0.860]	1.190 [0.735, 1.585]	<0.0001
Pod3 TP, (median [IQR])	62.200 [58.940, 65.150]	62.090 [58.840, 65.130]	62.540 [59.685, 65.390]	0.2822
Pod3 PAB, (median [IQR])	153.425 [128.675, 177.947]	153.960 [128.450, 177.050]	148.870 [128.750, 178.050]	0.6646
Pod3 TRF, (median [IQR])	2.110 [1.870, 2.420]	2.250 [2.040, 2.490]	1.730 [1.580, 1.830]	<0.0001
Pod3 PNI, (median [IQR])	48.250 [43.425, 52.470]	50.310 [45.370, 53.320]	43.200 [39.190, 46.700]	<0.0001
T, *n* (%)				0.3431
1	100 (24.75)	80 (26.58)	20 (19.42)	
2	50 (12.38)	35 (11.63)	15 (14.56)	
3	194 (48.02)	145 (48.17)	49 (47.57)	
4	60 (14.85)	41 (13.62)	19 (18.45)	
N, *n* (%)				0.8792
0	158 (39.11)	119 (39.53)	39 (37.86)	
1	64 (15.84)	49 (16.28)	15 (14.56)	
2	76 (18.81)	54 (17.94)	22 (21.36)	
3	106 (26.24)	79 (26.25)	27 (26.21)	
M, *n* (%)				0.9776
0	398 (98.51)	296 (98.34)	102 (99.03)	
1	6 (1.49)	5 (1.66)	1 (0.97)	
AJCC stage, *n* (%)				0.3976
I	112 (27.72)	87 (28.90)	25 (24.27)	
II	120 (29.70)	89 (29.57)	31 (30.10)	
III	161 (39.85)	115 (38.21)	46 (44.66)	
IV	11 (2.72)	10 (3.32)	1 (0.97)	
Depth of infiltration, *n* (%)				0.3106
mucosal layer	29 (7.18)	23 (7.64)	6 (5.83)	
submucosa	55 (13.61)	46 (15.28)	9 (8.74)	
muscle layer	92 (22.77)	68 (22.59)	24 (23.30)	
serous membrane layer	228 (56.44)	164 (54.49)	64 (62.14)	
Pod1 pain score, *n* (%)				<0.0001
1–3	262 (64.85)	215 (71.43)	47 (45.63)	
4–6	129 (31.93)	85 (28.24)	44 (42.72)	
7–10	13 (3.22)	1 (0.33)	12 (11.65)	
Exhaust time, h (median [IQR])	87.000 [68.750, 120.000]	87.000 [58.000, 96.000]	144.000 [99.000, 172.000]	<0.0001
Complication, n (%)				<0.0001
No	365 (90.35)	290 (96.35)	75 (72.82)	
Yes	39 (9.65)	11 (3.65)	28 (27.18)	

*Pre-WBC*, preoperative WBC; *Pre-Hb*, preoperative Hb; *Pre Neutrophil percentage*, preoperative neutrophil percentage; *pre-ALB*, preoperative ALB; *pre-NLR*, preoperative NLR; *pre-CAR*, preoperative CAR; *pre-TP*, preoperative TP; *pre-PAB*, preoperative PAB; *pre-TRF*, preoperative TRF; *pre-PNI*, preoperative PNI; *Pod1 WBC*, postoperative day 1 WBC; *Pod1 Hb*, postoperative day 1 Hb; *Pod1 Neutrophil*, percentage postoperative day 1 neutrophil percentage; *Pod1 ALB*, postoperative day 1 ALB; *Pod1 NLR*, postoperative day 1 NLR; *Pod1 CAR*, postoperative day 1 CAR; *Pod1 TP*, postoperative day 1 TP;*Pod1 PAB*, postoperative day 1 PAB; *Pod1 TRF*, postoperative day 1 TRF; *Pod1 PNI*, postoperative day 1 PNI; *Pod3 WBC*, postoperative day 3 WBC; *Pod3 Hb*, postoperative day 3 Hb; *Pod3 neutrophil percentage*, postoperative day 3 neutrophil percentage; *Pod3 ALB*, postoperative day 3 ALB; *Pod3 NLR*, postoperative day 3 NLR; *Pod3 CAR*, postoperative day 3 CAR; *Pod3 TP*, postoperative day 3 TP; *Pod3 PAB*, postoperative day 3 PAB; *Pod3 TRF*, postoperative day 3 TRF; *Pod3 PNI*, postoperative day 3 PNI; *AJCC stage*, American Joint Committee on Cancer Stage, and Pod1 pain score.

### Nomogram construction

**Figure 3:**
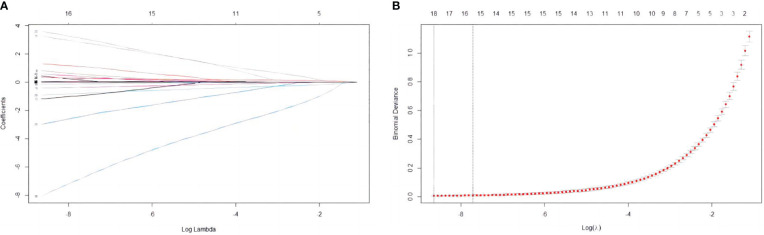
Variable selection based on the LASSO regression model. **(A)** The LASSO model was validated five times using minimum criteria to determine the best parameter (lambda). **(B)** LASSO coefficient profiles for the 15 features were plotted against log(lambda) sequences.

**Figure 4:**
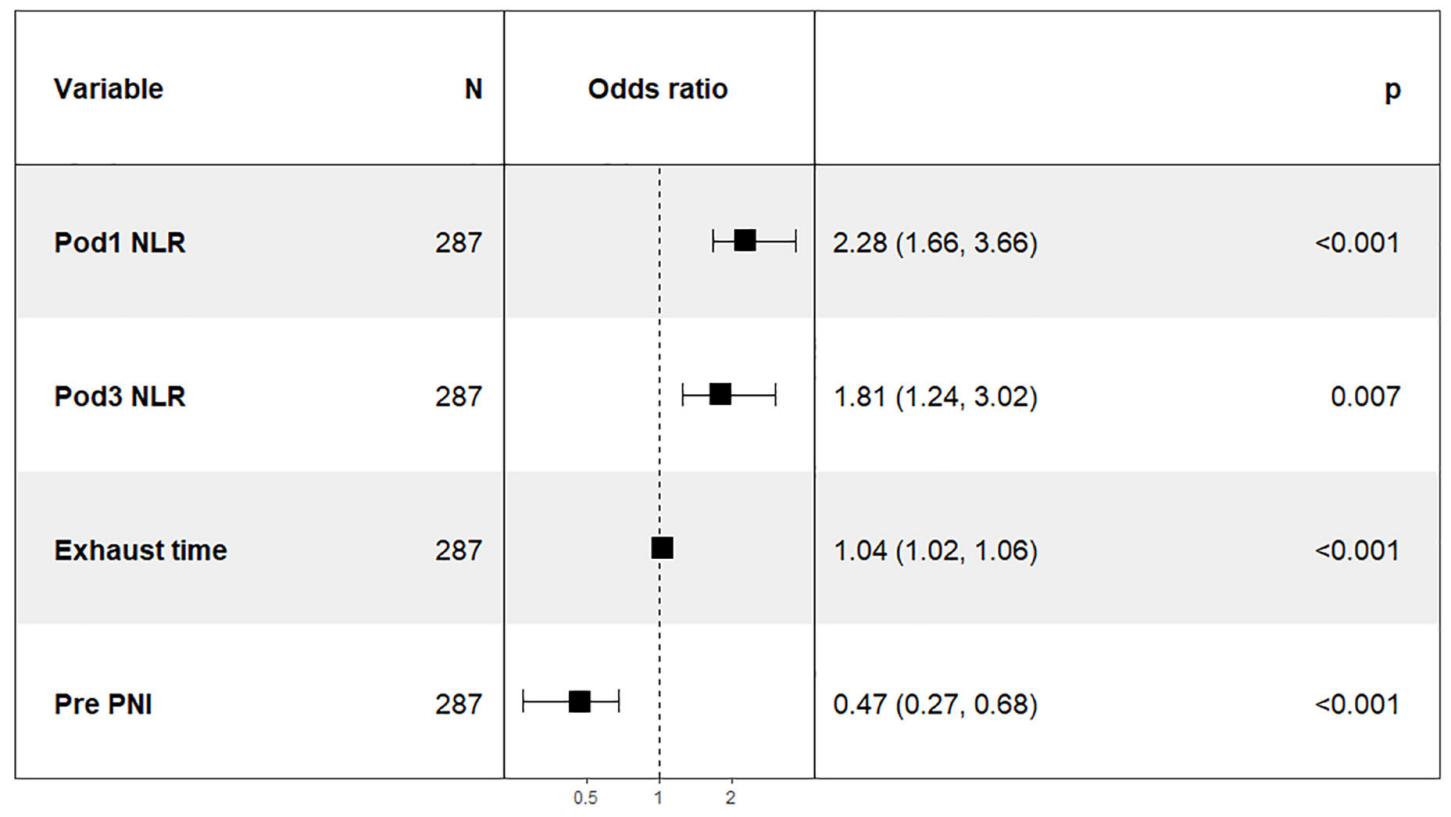
Logistic regression analysis of the predictors of prolonged PLOS.

**Figure 5:**
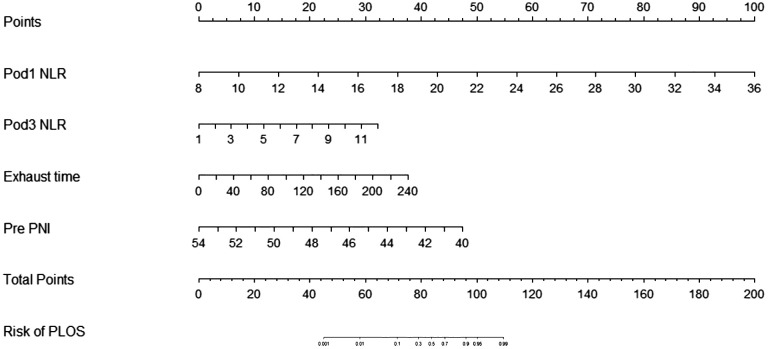
Risk factors for the Pod1 NLR, Pod3 NLR, Pre PNI and the exhaust time to complete the nomogram prediction model.

In this study, 47 variables obtained from clinical symptoms and laboratory tests were analyzed. To prevent overfitting and find the best penalty coefficient, lambda min was used as a criterion for selecting variables. Lambda represents the minimum mean square error. Using lambda min, we identified 15 variables with coefficients that were not zero (**Figure 3**). The 15 predictors were then used in a logistic regression analysis to create a prediction model. The results revealed that the pod1 NLR [OR=2.28, 95% CI (1.66, 3.66), *P*<0.001], pod3 NLR [OR=1.81, 95% CI (1.24, 3.02), *P*=0.007], exhaust time [OR=1.04, 95% CI (1.02, 1.06), *P*<0.001] and pre-PNI [OR=0.47, 95% CI (0.27, 0.68), *P*<0.001] were risk factors for prolonged PLOS (**Figure 4**). These variables were subsequently integrated into the prediction model to generate a risk nomogram for prolonged PLOS after gastrointestinal cancer surgery (**Figure 5**).

### Calibration and verification of the nomogram prediction model

**Figure 6:**
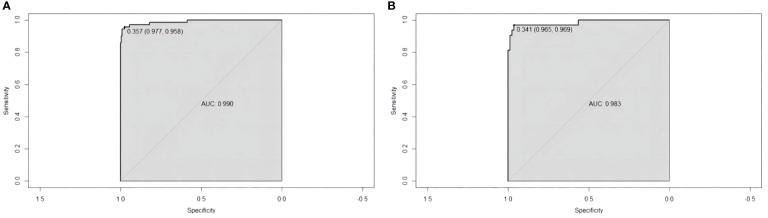
ROC curves for evaluating the reliability of the prediction model. **(A)** Training cohort ROC curve. **(B)** Validation cohort ROC curve.

**Figure 7:**
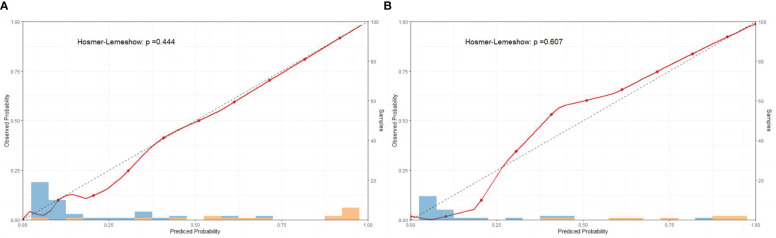
Calibration curves of the nomograms for the training and validation sets. **(A)** Calibration curve of training set; **(B)** Calibration curve of validation set.

**Figure 8:**
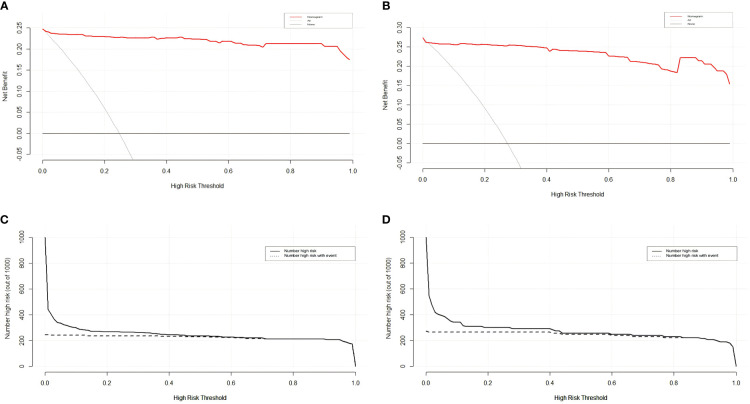
DCA and CICA of the nomograms for the training and validation sets on the basis of the risk stratification nomogram. **(A, C)** DCA and CICA curves of the training set. **(B, D)** DCA and CICA curves of the validation set.

The sensitivity and specificity of the prediction model were evaluated via ROC curve analysis. The AUC for the training set was 0.990, with a best cut-off value of 0.357. The model achieved a sensitivity of 0.958 and a specificity of 0.977. For the validation set, the AUC was 0.983, with the best cut-off value of 0.341. Similarly, the sensitivity and specificity were 0.969 and 0.965, respectively. The results indicate that our established model has high predictive accuracy (**Figure 6**). To calibrate the prediction model, a calibration chart was created. The p values obtained from the Hosmer–Lemeshow test for the training and validation sets were 0.444 and 0.607, respectively. These results suggest that there is no significant difference between the predicted and observed values. Therefore, the premeasured model demonstrates good calibration capability. The calibration curve results demonstrate a high degree of consistency between the actual predicted curve and the simulated predicted curve (**Figure 7**). Moreover, DCA and CICA revealed that the nomogram yielded superior net benefits in predicting the risk of prolonged discharge after surgery for GC patients (**Figure 8**).

**Figure 9:**
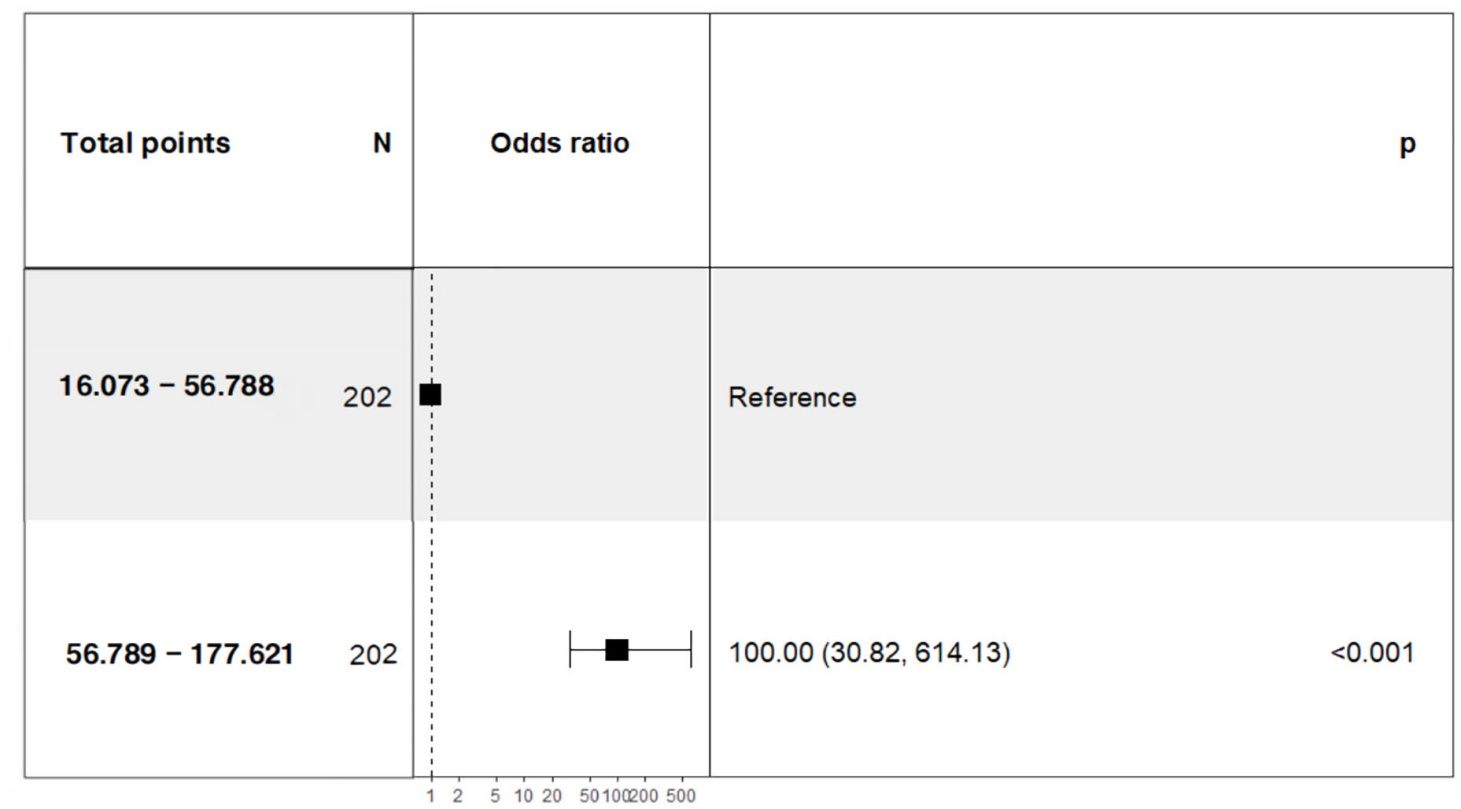
Correlations between the total points of the nomogram and prolonged PLOS.

To further investigate the correlation between the total score of the column chart and the risk of prolonged PLOS, we assigned scores to each independent factor in the column chart we constructed. We then calculated the total score for each patient on the basis of the variable score and divided the patients who underwent GC surgery into two groups: a low-risk group with a score of 56.788 or less and a high-risk group with a score greater than 56.789. Our analysis revealed a significant difference in the PLOS between high-risk patients (56.789–177.621; OR= 100.00; 95% CI = 30.82–614.13) and low-risk patients (**Figure 9**).

## Discussion

The PLOS is a crucial quality indicator for assessing the need for GC surgery. A well-moderated reduction in hospital stay after surgery helps maximize the effective utilization of medical resources. ERAS is a perioperative treatment approach that has emerged from evidence-based medical research. The primary objective of the ERAS approach is to optimize treatment methods and minimize surgical patients’ hospitalization duration, thereby reducing medical expenses. The PLOS has emerged as a prominent tool for assessing the efficacy of the ERAS approach (15). Despite considerable progress in surgical techniques for the treatment of GC, delayed discharge remains common because several factors affect postoperative recovery. The median PLOS for the patients in this study was 8 days. Multiple studies have demonstrated that the median duration of postoperative hospital stay could be reduced to 6–7 days by implementing ERAS in conjunction with robotic gastric cancer surgery (16). In China, it is hypothesized that the scarcity of rehabilitation hospitals and the unfavorable state of the national economy restrict most patients from completing their follow-up rehabilitation solely within hospitals, consequently leading to extended hospital stays. As a result, it is necessary to construct a predictive model via logistic regression to analyze the risk factors for long-term PLOS in patients with GC and implement measures to minimize delayed discharge.

A nomogram is a statistical modeling tool that visually represents the functional relationships between multiple independent variables within a rectangular coordinate system, utilizing nonintersecting line segments. According to multivariate analysis, each independent risk factor can be assigned a respective score, and the cumulative total corresponds to the likelihood of an endpoint event, thereby allowing for the prediction of disease prognosis. This study describes the risk factors associated with an extended PLOS for GC patients. The findings revealed that the Pod1 NLR, Pod3 NLR, Pre PNI and exhaust time contributed to prolonged hospitalization following surgery for GC. These factors were subsequently included in the nomogram evaluation model.

The inflammatory response is an important factor affecting the early postoperative recovery of patients with gastrointestinal malignant tumors. The NLR is derived from routine blood analysis and is more objective and stable than are white blood cells, neutrophils, lymphocytes and other counts, with less interference (16). Zahorec (17) reported that the gradual increase in lymphocytes and the gradual decrease in neutrophils coincided with improvements in several major clinical states of stress and the systemic inflammatory response. In contrast, if the neutrophil count continues to increase and the lymphocyte count continues to decrease for approximately one week, more severe complications may occur. A study by Che Morales revealed that the NLR in patients with pulmonary infection was significantly greater than that in healthy people and could be used to evaluate the prognosis of patients with pulmonary infection (18). As a highly sensitive marker of the systemic inflammatory response and immune homeostasis, the NLR increases when the number of lymphocytes decreases and the number of neutrophils increases, resulting in immune imbalance *in vivo* (19). In recent years, studies have shown that the NLR is an ideal marker for clinical treatment and plays an important role in evaluating and predicting the mortality and survival rates of patients during hospitalization without assistance (20).

The nutritional status of patients is influenced by the intake, absorption, and utilization of nutrients in the body, which are in turn affected by the physiological and pathological conditions of the patient. Malnutrition can impact surgical duration, postoperative complication rates, length of hospital stay, quality of life, and even mortality rates among cancer patients (21, 22). The PNI, which is calculated from preoperative serum ALB levels and peripheral blood lymphocyte counts, serves as a valuable tool for assessing a patient’s nutritional status and immune function. It also aids in predicting surgical risk and making prognostic judgements. The role of the PNI in predicting outcomes for patients with malignant tumors has become an area of active research interest (23, 24). This study revealed that low PNI values were associated with prolonged PLOS following gastric cancer surgery—a finding that is consistent with previous research results (25, 26). While there is currently no universally defined optimal critical value for the PNI, a comprehensive review of the current literature suggests that the optimal critical value falls within the range of 45–49.7 (27, 28). A meta-analysis involving 3,396 gastric cancer patients conducted by Yang et al. demonstrated that a low PNI was significantly correlated with prolonged postoperative hospitalization and an increased incidence of postoperative complications (OR=1.74, 95% CI=1.41–2.16; *P*<0.01) (29). The underlying reasons may be that PNI values are derived from peripheral blood lymphocyte counts and serum albumin levels—whereby albumin helps maintain plasma colloid osmotic pressure while alleviating immune responses—and that lymphocyte counts objectively reflect immune levels within the body. Patients with lower PNI levels may experience postoperative hypoalbuminaemia, leading to anastomotic oedema and poor healing outcomes at surgical sites—potentially resulting in complications such as anastomotic fistulae or intra-abdominal infections due to compromised immunity. Given these findings, clinicians should thoroughly evaluate patient nutrition prior to surgery; early intervention aimed at adjusting low PNI levels could reduce infectious complications after surgery, thereby promoting rapid recovery. For patients with lower PNI values before surgery, nutritional supplementation should be considered. Furthermore, patient prognosis after gastric cancer surgery can be effectively evaluated on the basis of preoperative PNI values. Therefore, a patient’s PNI value can be recorded as a routine item during clinical practice to guide treatment planning and serve as one factor used when evaluating prognosis. In addition, early gastrointestinal exhaustion is an important indicator of whether patients recover early. A shorter exhaust time and defecation duration indicate normal recovery of digestive tract function and fewer complications (30).

In conclusion, developing a nomogram offers clinicians a valuable tool for guiding therapeutic strategy decision-making and creating personalized treatment plans for patients undergoing GC surgery. Active multidisciplinary treatment and comprehensive treatment, which include acupuncture and moxibustion, have shown substantial potential for improving the prognosis of patients with high-risk factors. Nevertheless, it is essential to acknowledge the limitations of this study. First, as a single-center retrospective analysis, we excluded patients with incomplete information, resulting in an insufficient sample size and the potential for introducing selection bias. Furthermore, we did not include specific postgastric cancer surgery gastrointestinal function indicators, such as gastrin. Moreover, multiple factors can impact hematological examination results. Hence, further clinical research is necessary to provide additional validation and support in the future.

## Data availability statement

The raw data supporting the conclusions of this article will be made available by the authors, without undue reservation.

## Ethics statement

The studies involving humans were approved by Ethics Committees of Affiliated Hospital of Nanjing University of Chinese Medicine. The studies were conducted in accordance with the local legislation and institutional requirements. The participants provided their written informed consent to participate in this study.

## Author contributions

XZ: Data curation, Formal analysis, Methodology, Visualization, Writing – original draft, Writing – review & editing. XW: Data curation, Writing – original draft. SL: Data curation, Investigation, Writing – review & editing. WS: Data curation, Investigation, Writing – original draft. CW: Investigation, Writing – original draft. WC1: Formal Analysis, Investigation, Software, Writing – original draft. GG: Software, Supervision, Writing – review & editing. GW: Project administration, Writing – review & editing. WC2: Methodology, Supervision, Writing – review & editing. MS: Writing – review & editing. ZD: Conceptualization, Data curation, Formal analysis, Investigation, Supervision, Validation, Writing – review & editing. ZJ: Conceptualization, Funding acquisition, Investigation, Resources, Supervision, Writing – review & editing.
